# Supplementary material for: Safety of a Sustainably Produced, Bioengineered, Nature-Identical Salidroside Compound
Source: Nutrients. 2022 Jun 1;14(11):2330. doi: 10.3390/nu14112330 (PMC9183058; doi:10.3390/nu14112330)
Supplement: Supplementary file 1 [file nutrients-14-02330-s001.zip › nutrients-1734539-supplementary.pdf]

Supplementary Table 1. Reverse mutation assay of salidroside in *Salmonella typhimurium* and *Esherishia coli*: mean number of revertants/plate

Concentration (µg/plate)	TA98		TA100		TA1535		TA1537		*E. coli* WP2 uvrA	
	-S9	+S9	-S9	+S9	-S9	+S9	-S9	+S9	-S9	+S9
**Experiment 1 (mean±standard deviation)**										
0[a]	20 ± 3.1	21 ± 0.6	113 ± 18.2	109 ± 18.9	11 ± 0.6	11 ± 1.5	9 ± 1.5	11 ± 1.2	42 ± 8.5	50 ± 6.5
1.58	22 ± 3.1	24 ± 4.6	114 ± 10.0	112 ± 14.4	10 ± 3.1	12 ± 1.0	8 ± 1.5	10 ± 3.0	47 ± 2.1	48 ± 7.5
5.0	21 ± 1.7	23 ± 5.7	119 ± 3.1	112 ± 3.8	12 ± 1.7	11 ± 1.0	13 ± 0.6	12 ± 4.0	32 ± 1.7	54 ± 8.5
15.8	22 ± 8.9	29 ± 1.5	108 ± 2.6	121 ± 6.5	10 ± 2.5	11 ± 2.3	9 ± 3.5	9 ± 1.2	37 ± 2.6	47 ± 5.0
50	23 ± 5.5	24 ± 3.1	122 ± 4.6	107 ± 13.9	11 ± 4.2	13 ± 2.3	9 ± 0.6	10 ± 3.2	43 ± 6.4	46 ± 12.1
158	24 ± 3.5	21 ± 1.0	110 ± 4.2	105 ± 9.7	13 ± 4.2	12 ± 3.5	10 ± 2.3	10 ± 2.6	33 ±1.5	48 ± 8.5
500	22 ± 1.2	27 ± 2.3	104 ± 6.7	103 ± 12.1	11 ± 3.8	10 ± 3.0	9 ± 1.7	12 ± 1.7	39 ± 2.6	55 ± 2.6
1580	22 ± 1.0	28 ± 3.2	116 ± 6.1	111 ± 2.5	10 ± 2.5	10 ± 4.4	9 ± 1.0	11 ± 1.0	38 ± 1.4	51 ± 7.9
5000	21 ± 4.0	26 ± 2.1	115 ± 10.0	116 ± 5.5	14 ± 1.2	10 ± 4.0	11 ± 2.9	10 ± 1.5	37 ± 6.1	43 ± 2.1
Positive control	223 ± 65.0 [b]	141 ± 26.1[c]	442 ± 39.9[d]	779 ± 50.8[c]	557 ± 27.7[d]	367 ± 9.3 [e]	230± 16.1 [f]	261 ± 20.0 [e]	950 ± 26.2 [g]	188 ± 0.6 [e]
**Experiment 2 (mean±standard deviation)**										
0[a]	23 ± 6.7	24 ± 4.0	107 ± 7.0	108 ± 7.6	10 ± 1.2	11 ± 1.7	13 ± 0.6	12 ± 3.1	37 ± 8.7	55 ± 6.1
1.58	19 ± 1.2	23 ± 2.5	119 ± 4.5	116 ± 7.8	10 ± 1.2	10 ± 2.5	15 ± 3.6	12 ± 2.0	37 ± 6.5	45 ± 5.5
5.0	20 ± 2.1	27 ± 1.5	115 ± 8.5	125 ± 4.0	10 ± 3.2	12 ± 3.8	12 ± 4.5	8 ± 1.5	34 ± 5.6	42 ± 6.5
15.8	25 ± 5.0	27 ± 3.2	118 ± 7.6	113 ± 8.2	13 ± 3.5	9 ± 1.0	10 ± 3.1	13 ± 6.8	35 ± 7.6	49 ±1.5
50	21 ± 2.3	23 ± 1.7	113 ± 13.2	114 ± 12.2	10 ± 2.1	12 ± 0.0	14 ± 1.5	14 ± 1.0	40 ± 1.5	50 ± 5.7
158	19 ± 1.2	23 ± 1.0	122 ± 7.4	113 ± 11.1	9 ± 2.5	9 ± 4.4	17 ± 0.6	14 ± 2.5	34 ± 5.3	46 ± 4.6
500	20 ± 1.5	24 ± 0.6	117 ± 10.3	121 ± 3.0	11 ± 0.6	10 ± 1.7	12 ± 1.2	14 ± 2.5	36 ± 10.4	42 ± 6.0
1580	19 ± 4.9	25 ± 2.6	95 ± 5.5	97 ± 14.3	10 ± 3.6	8 ± 2.0	10 ± 2.1	10 ± 2.0	31 ± 3.5	31 ± 1.7
5000	21 ± 2.5	22 ± 2.6	120 ± 2.1	113 ± 6.6	12 ± 1.5	10 ± 1.0	11 ± 2.1	10 ± 1.7	30 ± 3.1	41 ± 3.8
Positive control	265 ± 84.1 [b]	179 ± 5.1[c]	515 ± 27.6[d]	798 ± 22.4[c]	491 ± 7.0[d]	400 ± 14.2 [e]	478 ± 45.4 [f]	262 ± 40.9 [e]	891 ± 15.4 [g]	120 ± 15.2 [e]

Substance was tested using the standardized plate incorporation assay (Experiment 1) and the pre-incubation method (Experiment 2). Results are means of three replicates per test condition.
[a]Sterile water; [b]2-nitrofluorene; [c]benzo{a}pyrene; [d]Sodium azide; [e]2-aminoanthracene; [f]9-aminoacridine hydrochloride monohydrate; [g]4-nitroquinoline N-oxide

Supplementary Table 2. Mean Body Weights for the 7-day Range-finding Study

Day(s) Relative to Start Date	Control	500 mg/kg bw/day	1000 mg/kg bw/day	2000 mg/kg bw/day
**Males (g)**				
1	216.4 ± 16.7	217.4 ± 20.4	216.2 ± 15.8	218.8 ± 18.6
7	267.2 ± 14.2	269.0 ± 25.1	260.4 ± 16.8	270.0 ± 24.4
8	275.8 ± 12.8	277.6 ± 24.2	264.2 ± 17.1	279.6 ± 26.3
**Females (g)**				

1	191.6 ± 10.5	191.8 ± 13.1	189.4 ± 13.1	193.4 ± 16.7
7	208.4 ± 13.2	215.4 ± 11.7	208.6 ± 13.7	217.0 ± 13.8
8	209.2 ± 11.1	217.0 ± 14.1	208.6 ± 13.0	216.6 ± 11.4

n= 5/sex/group; mean ± standard deviation; bw = body weight; g = grams; kg = kilograms; mg = milligrams

Supplementary Table 3. Mean Body Weight Gain for the 7-day Range-finding Study

Day(s) Relative to Start Date	Control	500 mg/kg bw/day	1000 mg/kg bw/day	2000 mg/kg bw/day
		**Males (g)**		
1-7	8.47 ± 1.81	8.60 ± 1.06	7.37 ± 0.34	8.53 ± 1.24
7-8	8.60 ± 2.88	8.60 ± 2.30	3.80 ± 1.48*	9.60 ± 3.05
		**Females (g)**		
1-7	2.80 ± 1.16	3.93 ± 0.84	3.20 ± 0.62	3.93 ± 0.73
7-8	0.80 ± 3.49	1.60 ± 4.72	0.00 ± 1.00	-0.40 ± 4.34

n= 5/sex/group; mean ± standard deviation; bw = body weight; g = grams; kg = kilograms; mg = milligrams; * Anova & Dunnett = $p<0.05$

Supplementary Table 4. Mean Daily Food Consumption for the 7-day Range Finding Study

Day(s) Relative to Start Date	Control	500 mg/kg bw/day	1000 mg/kg bw/day	2000 mg/kg bw/day
		**Males (g)**		
1-7	25.77 ± 1.58	25.83 ± 0.76	23.90 ± 0.55	26.03 ± 2.08
		**Females (g)**		
1-7	16.53 ± 0.05	18.17 ± 0.30*	16.80 ± 0.49	18.53 ± 0.72*

n= 5/sex/group; mean ± standard deviation; bw = body weight; g = grams; kg = kilograms; mg = milligrams; * Anova & Dunnett (rank) = $p<0.001$,
